# Mental health and sleep quality of low-income mothers of one-year-olds during the COVID-19 pandemic

**DOI:** 10.1002/imhj.22074

**Published:** 2023-07

**Authors:** Elizabeth M. Premo, Katherine A. Magnuson, Nicole E. Lorenzo, Nathan A. Fox, Kimberly G. Noble

**Affiliations:** 1 Sandra Rosenbaum School of Social Work, University of Wisconsin-Madison, Madison, Wisconsin, USA; 2 Department of Human Development and Quantitative Methodology, University of Maryland, College Park, Maryland, USA; 3 Department of Biobehavioral Sciences and Human Development, Teachers College, Columbia University, New York City, New York, USA

**Keywords:** anxiety, COVID-19 pandemic, depression, poverty, sleep quality

## Abstract

The COVID-19 pandemic and subsequent social restrictions created an unprecedented context for families raising young children. Although studies have documented detrimental effects of the pandemic on maternal well-being, less is known about how the pandemic specifically impacted low-income mothers. We examined depression, anxiety, and sleep quality among low-income mothers of one-year-olds during the early months of the pandemic using data from the Baby’s First Years study. Focusing on the control group (*n* = 547), we compared mothers interviewed before March 14th, 2020 (*n* = 342) to mothers interviewed between March 14th and June 30th, 2020 (*n* = 205) to determine whether the pandemic was associated with differences in mental health and sleep quality. Mothers were recruited from four cities in the United States, and most of the sample identified as Hispanic (42.2%) or Black, non-Hispanic (38.6%). We found that mothers interviewed during the pandemic reported better mental health and sleep quality. While we cannot speak to longer-term impacts of the pandemic, it is possible low-income mothers experienced relief from daily stressors during the initial shelter-in-place orders, which may have led to improvements in well-being. These results have implications for understanding how complex life stressors influence mental health and sleep quality among low-income mothers raising young children.

## 1 INTRODUCTION

Mothers of young children are at an elevated risk of experiencing issues with mental health and sleep quality, especially in the first year after childbirth (Biaggi et al., 2016; O’Hara & Swain, 1996; Park et al., 2013). Low-income mothers are particularly vulnerable to lower quality mental health and sleep because of increased economic and social stressors (Doering et al., 2017; Lee & Gay, 2011; Rich-Edwards et al., 2006). Depression, anxiety, and sleep problems can negatively impact mothers’ overall well-being and their children’s physical, cognitive, and emotional development (Beck, 1998; Feldman et al., 2009; Insana et al., 2013; Piteo et al., 2013). Therefore, maternal mental health and sleep quality are important public health issues.

The early phase of the COVID-19 pandemic created unprecedented disruptions in the lives of families raising young children. Shelter-in-place orders, increased health concerns, unemployment, income losses, and childcare and school closures affected families’ lives. At the onset of the COVID-19 pandemic, researchers, policymakers, and practitioners were quick to express concerns about maternal well-being due to the increased stressors, economic hardships, and social isolation that many families were facing (Thapa et al., 2020). While the pandemic altered life for everyone, there were concerns that families living in poverty and communities of color may have been disproportionately harmed (Condon et al., 2020; Taylor et al., 2020).

The current study used a unique, prospective longitudinal dataset to consider whether the mental health and sleep quality of low-income mothers with young children were harmed by the early phase of the COVID-19 pandemic when shelter-in-place orders were widespread. We focused on mothers’ subjective experiences of their well-being by examining their self-reports of depression, anxiety, and sleep quality. Our sample allowed us to shed light on an overlooked group of mothers, specifically those who have young children and low incomes. The mothers in this study all reported household incomes below the federal poverty line (FPL) at the time of their child’s birth, though their incomes fluctuated slightly over the first year of their child’s life.^1^ Through this work, we filled an important gap in the literature by examining whether low-income mothers’ experiences of depression, anxiety, and sleep quality differed before and after the onset of the pandemic.

### 1.1 | Social determinants of health framework

The social determinants of health framework describes how societal factors and the social environment impact the physical and mental health of marginalized groups (Adler & Rehkopf, 2008; Adler & Stuart, 2010; Brondolo et al., 2009). This theoretical model suggests that low incomes can negatively impact the mental and physical well-being of mothers raising young children because of the social stressors related to poverty, including financial hardship, food insecurity, lack of partner or familial support, and childcare and work-related stressors (Linares et al., 2020; Manuel et al., 2012). Though experienced on an individual level, many of these threats to health are rooted in systemic and structural inequities that both historically and currently harm low-income mothers (Crear-Perry et al., 2021; Woolf & Braveman, 2011).

Given the potential increase in social stressors during the COVID-19 pandemic, including health concerns, economic losses, and social isolation, this framework might predict negative impacts of the pandemic on low-income mothers’ well-being, especially considering these mothers were already experiencing insufficient resources and low social support. Although this was an understandable concern, there were also reasons to be skeptical of this prediction. For example, it was also possible that the social context of the early COVID-19 pandemic led to low-income mothers having more support at home, expanded community resources (e.g., food pantries), increased time flexibility, decreased work-family challenges, and a more responsive social safety net. These factors could have led to temporary reductions in stress and thus improvements in the quality of life for this specific group of mothers.

### 1.2 | Mental health and sleep quality among low-income mothers

While the early years of a child’s life are a source of joy for most parents, it is also a developmental period that is associated with increased vulnerability to mental health issues, especially depression and anxiety. Approximately 17% of mothers experience depression in the first year of their child’s life, while 15% experience anxiety (Dennis et al., 2017; Shorey et al., 2018). Sleep quality is commonly discussed alongside depression and anxiety because sleep is a crucial component of psychosocial well-being (Goldstein & Walker, 2014). Sleep quality refers to how satisfied someone is with their overall sleeping experiences, whereas sleep problems focus on potential sources or reasons for dissatisfaction, including difficulty falling asleep or waking while sleeping.

The connection between sleep quality and mental health is especially salient for mothers of young children, who experience profound changes to their sleep patterns after having children. A survey of mothers with children ages zero to five found that more than half of mothers experienced poor quality sleep and frequently experienced symptoms related to insomnia, such as difficulty falling asleep and waking up in the night (Mindell et al., 2013). Sleep disturbances are strongly correlated with psychological distress and daily mood for adults in general (Bower et al., 2010), and for mothers of young children, their subjective reports of poor sleep quality are strongly associated with worse symptoms of depression and anxiety (Bei et al., 2010; Gonzalez-Mesa et al., 2019; Okun et al., 2018).

Although mental health and sleep quality are concerns for all parents of young children, low-income mothers report higher rates of depression and anxiety as well as lower quality sleep compared with the average mother (Doering et al., 2017; Lee & Gay, 2011; Rich-Edwards et al., 2006). Rates of chronic depression among poor mothers of young children are almost three times higher compared to their non-poor counterparts (Wang et al., 2011). Similarly, Baer et al. (2012) found that poor mothers have greater odds of meeting the diagnostic criteria for generalized anxiety disorder, and they argue that these increased rates of anxiety reflect these mothers’ reactions to environ mental social conditions related to poverty. In a study of low-income mothers, more than 70% of women indicated having at least a little problem with sleep and about 25% reported poor or very poor sleep quality (Mersky et al., 2020). Overall, studies have established that low-income mothers have elevated rates of depression and anxiety, and there is emerging evidence that they experience more issues with sleep as well.

The relationship between income and maternal well-being is complex, and several factors contribute to higher rates of depression, anxiety, and sleep problems among low-income mothers. Recent studies have explored how differences in postpartum health care contribute to worse mental health for low-income mothers, including a lack of mental health screening, mothers’ distrust of providers, and experiences of racism (Abrams et al., 2009; Klawetter et al., 2020; Murphy et al., 2016; Orengo-Aguayo & Segre, 2016). Other economic and social factors are also associated with worse mental health outcomes for mothers experiencing poverty, including unavailable or inadequate childcare, limited partner and social support, and inflexible employment schedules and obligations (Bryant et al., 2006; Corrigan et al., 2015; Letourneau et al., 2009; Smith et al., 2000). Similarly, differences in sleep quality among low-income mothers could be connected to ecological factors, such as adverse life events, nonstandard work hours, and chaotic or noisy households. A study of low-income African American mothers found that poor sleep quality was not attributable to their babies’ sleep patterns; instead, these mothers described psychological (e.g., emotional distress, worries) and environmental factors (e.g., work commitments, distractions in the home) that negatively influenced their sleep quality (Zambrano et al., 2016). Ultimately, differences in social environments may be important for understanding mental health and sleep outcomes for low-income mothers.

### 1.3 | The impact of the COVID-19 pandemic on maternal well-being

The COVID-19 pandemic upended many aspects of family life. Yet, its effects might have been especially pronounced for families of young children. Parents faced shelter-in-place orders that might have limited their ability to seek support from extended family and friends, while also increasing parents’ time spent at home with their children. In addition, school and childcare closures may have disrupted family routines and employment schedules. Given that depression, anxiety, and sleep quality are sensitive to changes in life stressors, these disruptions may have impacted maternal well-being.

Several studies have examined the detrimental effects of the COVID-19 pandemic on maternal mental health. An early meta-analysis compiled results from eight studies on depression and anxiety of pregnant or postpartum mothers during the pandemic (Hessami et al., 2020). They found that anxiety rates were significantly higher (.82 standardized mean difference on the State-Trait Anxiety Inventory) during the pandemic, but depression rates were not significantly higher. A key limitation of this meta-analysis was that many of the studies had methodological weaknesses. Specifically, the study samples were often comprised of volunteers recruited from social media advertisements and unsolicited emails. These convenience sampling strategies may lead to bias because mothers self-selected into the studies after the onset of the pandemic and are not representative of any specific population.

A subsequent international meta-analysis of 18 studies examined the prevalence of depression and anxiety symptoms among mothers of young children during the COVID-19 pandemic (Racine et al., 2022). On average, 26.9% of mothers had clinically elevated depressive symptoms and 41.9% had clinically elevated anxiety symptoms. Rates of depression and anxiety differed based on demographic factors. Depression and anxiety symptoms were higher among demographic groups that are more socially and economically advantaged. For example, studies of predominantly White mothers showed higher rates of clinically elevated depressive symptoms compared to studies with a higher percentage of racial and ethnic minority women. Likewise, clinically elevated anxiety symptoms were more common among samples with more educated mothers. Similarly, a study that explored the mental health of mothers during COVID-19 lockdowns in China, Italy, and the Netherlands found that higher family income, higher education, and older maternal age were all associated with worse mental health during the pandemic (Guo et al., 2021). These results suggest that marginalized families experienced fewer declines in mental health than other families during the pandemic, so we might expect low-income mothers (who are disproportionately likely to be racially minoritized, have lower education, and be younger) to show similar resiliency in terms of mental health during this time.

There has been little research on the impact of the COVID-19 pandemic on maternal sleep quality. Given studies suggesting that psychological distress increased during the pandemic, and psychological distress is often accompanied by low quality sleep, we might expect sleep quality to worsen as well. However, findings are mixed. Some studies find no overall differences in adult’s sleep quality (Gao & Scullin, 2020), and other studies find increases in sleep problems such as difficulty falling asleep (Hisler & Twenge, 2021). To date, there has been minimal research on self-reported perceptions of sleep quality among mothers of young children in the United States. For this population, it is also possible that shifts in daily routines that previously impeded sleep quality (e.g., stressors related to rigid work schedules, coordinating childcare, and long morning commutes) might have led to improved sleep quality during this time. The COVID-19 pandemic created a unique context of shelter-in-place orders and suspension of many daily activities, which might have altered both risk and protective factors related to sleep, especially for mothers raising young children.

### 1.4 | Unique experience of low-income mothers during the COVID-19 pandemic

Most studies on maternal mental health and sleep quality during the pandemic have used samples that were relatively economically privileged or failed to explore potential differences by income. Therefore, there is limited evidence on whether and how the pandemic has impacted families that were already experiencing poverty and economic hardship prior to the pandemic. One exception is a study by Gassman-Pines et al. (2020) that examined the effects of the pandemic on mood and sleep quality among low-wage service workers who had children aged 2–7 years old. The study collected daily survey data, which included a single item about negative mood and a single item about perceived negative sleep quality. The authors found that mood worsened for low-wage service workers during the initial weeks of the pandemic, but sleep quality was unaffected. Families who experienced multiple hardships related to the pandemic (e.g., job loss, income loss) had decreases in both mood and sleep quality. Of course, service workers were hit hard by the pandemic especially early on when little was known about how to protect against transmission. Given the challenges and risks with employment in the service sector, it is unclear whether these findings would extend to all low-income families, including those with younger children and those who were not employed in the service sector or at all. Indeed, low-income mothers with one-year-olds are less likely to be employed compared with mothers of older children (U.S. Department of Labor, 2019).

Silverman et al. (2020) provided a unique examination of how the COVID-19 pandemic affected postpartum mothers’ mental health and found positive impacts for mothers with low socioeconomic status^2^. Using patient data from postpartum mothers in New York City, they found that mothers from lower socioeconomic backgrounds had significantly lower rates of postpartum depression during the social restrictions of the COVID-19 pandemic compared to before the onset of the pandemic. This improvement in postpartum mood during the early phase of the pandemic was evident only among mothers with low socioeconomic status, with more economically advantaged mothers reporting no significant differences. The authors speculated that due to shelter in-place-orders, these mothers may have experienced fewer daily challenges during the pandemic compared to prior. Given its use of health record data, this study did not have detailed information on family income or other economic background characteristics. As a result, it is unclear how this might generalize to a more narrowly defined low-income or poverty population, but it at least suggests that negative impacts on mood may have been more selective than previously assumed.

As suggested by Silverman et al. (2020), it is possible that mothers who were already experiencing major economic stressors might have responded differently to the COVID-19 pandemic compared to families that were economically privileged. For example, economically privileged mothers who previously had stable childcare and flexible work arrangements may have had a larger shock at the onset of the pandemic when disruptions to their support systems and resources led to increased burdens of housework and childcare compared to their pre-pandemic lives. Among low-income parents who were already juggling daily demands that outweighed their resources, the early pandemic may have led to fewer of those disruptions and instead were experienced as a relief from their daily stressors and a welcomed opportunity to spend more time at home with their young children. Although Silverman and colleagues provide some evidence that low-income mothers experienced improvements in mood during the early phase of the pandemic, overall there is still limited and mixed evidence on mental health and sleep quality among low-income mothers of young children during the pandemic, which calls for additional research to explore the well-being of this vulnerable group.

### 1.5 | Current study

In this study, we compared self-reported depression, anxiety, and sleep quality of low-income mothers of one-year-olds before and during the COVID-19 pandemic. We did this using a sample of mothers interviewed for an ongoing study at the time of their child’s first birthday. For two thirds of the mothers this interview was conducted in-person prior to the pandemic (before March 14th, 2020), and for one third of the mothers it occurred over the phone during the early social restrictions of the COVID-19 pandemic (between March 14th, and June 30th, 2020). Because the timing of the child’s birthday is random, the mothers interviewed before and after the onset of the pandemic were expected to be similar except for their experiences of life during the COVID-19 pandemic and the modality of the interview. We used this variation in the timing of the interview to examine whether there were associations between the early phase of the pandemic and mothers’ self-reported mental health and sleep quality. Because most prior research found negative associations between the pandemic and maternal mental health and sleep quality, we hypothesized that mothers in this study would also have worse mental health and sleep quality during the pandemic compared to mothers interviewed prior to the pandemic.

We also explored whether demographic or family characteristics moderated the associations between the pandemic and mental health or sleep quality. Specifically, we tested whether the associations differed based on relationship status (married/cohabitating versus single), employment history (employed versus unemployed prior to the child’s birth), and mother’s race and ethnicity. We hypothesized that mental health and sleep quality would be better during the pandemic among mothers who were married or cohabitating with partners than mothers who were single. We also hypothesized that mothers who had a history of employment prior to the child’s birth would experience worst mental health and sleep quality during the pandemic than mothers who were unattached to the labor market. As an exploratory analysis, we also looked at differences by mother’s race and ethnicity, but we did not have a priori hypotheses about how race and ethnicity would moderate the relationships between the pandemic and mental health or sleep quality.

This study adds to our empirical understanding of low-income mothers’ unique experiences of depression, anxiety, and sleep quality during the early phase of the pandemic. Furthermore, the methodological strengths of this study—namely that it was a prospective sample recruited prior to the onset of the pandemic and was composed of a large and diverse set of low-income, urban mothers—help overcome the limitations of prior research.

## 2 | METHOD

### 2.1 | Data and sample

This study used data from the Baby’s First Years (BFY) project, which is a randomized controlled trial testing the impact of monthly cash gifts to low-income mothers during the first 76 months of their children’s lives. BFY enrolled 1000 mothers of newborns with household incomes below the federal poverty line from 12 local hospitals across four diverse U.S. sites shortly after their children were born between May 2018 and June of 2019. To enroll in the study, mothers needed to complete a screening interview to determine eligibility, participate in the baseline interview, and agree to receive a debit card with a monthly cash gift. The full set of inclusion criteria and details of the study design are available in Noble et al. (2021), but the key inclusion criteria were that the mother was of legal age; she self-reported a household income below the federal poverty threshold; the infant was in the well child nursery; and the mother spoke either English or Spanish. Mothers received monthly cash payments by debit card and the amount of the cash gift was randomized after enrollment into the longitudinal study. Mothers in the high cash gift (treatment) group (*n* = 400 in the full study) received a cash gift of $333 per month ($3996 per year), whereas mothers in the low cash gift (comparison) group (*n* = 600 in the full study) received a nominal monthly gift of $20 ($240 per year).

The current study used data from the Age-1 wave of interviews that took place between July 2019 and June 2020. The sample for this study was restricted to the comparison group only because mothers in the high cash group may have had differing experiences during the pandemic than the comparison group families, given their access to the larger unconditional monthly cash gifts. Of the 600 mothers who were assigned to the comparison condition, 548 completed the Age-1 survey (91.33% retention rate). Of the 52 (8.67%) comparison group mothers who did not complete the Age-1 survey, three were incarcerated, seven were no longer living with the child, one had a child who was deceased, 33 were not able to be located, six refused the interview, and two completed only minimal parts of the interview. Surveys were conducted in English (*n* = 408; 74.45%) or Spanish (*n* = 140; 25.55%) depending on the language preference of the participant.

To be included in the analytic sample for this study, mothers had to have valid data on at least one of our three dependent variables. Of the 548 mothers who completed the Age-1 survey, there were minimal missing data on our measures of depression, anxiety, and sleep quality. One mother had missing data on depression, anxiety, and sleep quality, and an additional mother had missing data on sleep quality only. Thus, our final analytic sample (*n* = 547) excluded one mother who had missing data on all three dependent measures. For our analyses on sleep quality, the sample size reduced by one (*n* = 546) to exclude the mother with missing data on this outcome.

To examine the associations between the COVID-19 pandemic and maternal mental health and sleep quality, we used an indicator to identify two cohorts of mothers based on whether their Age-1 interview occurred before the onset of the shelter-in-place orders (i.e., before March 14th, 2020) or during the social restrictions of the early phase of the pandemic (i.e., between March 14th and June 30th, 2020). This allowed us to compare the mental health and sleep quality of mothers who were interviewed before the pandemic (*n* = 342 of 545 that were available to be interviewed) to those who were interviewed after the onset of the pandemic (*n* = 205 of 256 who were not yet interviewed by March 14th) to determine if there were significant differences in their self-reported depression, anxiety, and sleep quality.

### 2.2 | Measures

#### 2.2.1 | COVID-19

The onset of the COVID-19 pandemic was operationalized by the date at which the study protocol switched from in-person data collection to telephone interviews due to shelter-in-place orders in all four sites. This binary indicator specified whether the interview took place before or after March 14th, 2020. Mothers in the pre-pandemic group were interviewed between July 26th, 2019 and March 13th, 2020, and mothers in the during-pandemic group were interviewed between March 17th, 2020 and June 30th, 2020.

Our measure of being interviewed during the COVID-19 pandemic is confounded with the mode of the interview. While this might be a concern, prior studies have found that individuals report similar levels of depressive and anxiety symptoms whether being asked by someone in-person or over the phone (Aneshensel et al., 1982; Paulsen et al., 1988; Rohde et al., 1997). For this reason, we attributed differences in responses between groups to be due to the experiences of the pandemic and related shelter-in-place orders rather than interview modality.

#### 2.2.2 | Maternal depression

Depression was measured using the self-reported Patient Health Questionnaire-8 (PHQ-8), which is an additive index of eight items measured on a four-point Likert scale (0: not at all; 1: several days; 2: more than half of days; 3: every day; Kroenke & Spitzer, 2002). The eight items represent common symptoms of major depressive disorder, including little interest or pleasure doing things, trouble concentrating, and feeling down, depressed, or hopeless. Scores were truncated to the 99th percentile, and the scale had acceptable internal reliability (*a* = .70). On average, rates of maternal depression were moderate to low (*M* = 3.70, SD = 4.01), with 9.87% of mothers meeting criteria for moderate to severe depression (i.e., 10 or greater on the PHQ-8).

#### 2.2.3 | Maternal anxiety

Anxiety was measured using the self-reported Beck Anxiety Inventory (BAI), which is an additive index of 21 items measured on a four-point Likert scale (0: not at all; 1: mildly; 2: moderately; 3: severely bothersome; Steer & Beck, 1997). This scale includes both psychological (e.g., nervous, scared) and somatic (e.g., hands trembling, heart pounding) symptoms of anxiety. Notably, this scale has greater emphasis on physiological symptoms associated with panic than symptoms of generalized worry. Scores were truncated to the 99th percentile, and the scale had good internal reliability (*a* = .89). On average, mothers reported moderate to low levels of anxiety (*M* = 4.52; SD = 6.32), with 7.13% of mothers meeting criteria for moderate to severe anxiety (i.e., 16 or greater on the BAI).

#### 2.2.4 | Sleep quality

Sleep quality was measured using a self-reported additive index of three items, each measured on a five-point Likert scale (0: very poor/not at all; 5: very good/very much). The items were adapted from the Patient-Reported Outcomes Measurement Information System (PROMIS^™^) Short-Form (Yu et al., 2012). Mothers were asked to rate: (1) quality of sleep; (2) difficulty falling asleep; and (3) feeling tired. Two of the items were reverse scored so that higher scores on the index indicated better sleep quality. Scores were truncated to the 99th percentile, and the scale had acceptable internal reliability (*a* = .74). On average, mothers reported fair to good sleep quality (*M* = 10.77, SD = 2.94).

#### 2.2.5 | Baseline control variables

A series of variables that were self-reported by mothers on the baseline survey at the time of initial recruitment (i.e., approximately one year prior to the current wave of data collection, between July of 2018 and May 2019) were used to analyze and address baseline equivalence between the two groups. Given that the child’s birthday was used as the target for the interview date, in theory the two groups of mothers should be equivalent on baseline characteristic since birth dates are distributed at random. However, it is possible that baseline differences may have occurred if there were differences among those who were assigned to complete the interview before the pandemic and did so, compared with those who were assigned to complete the interview before the pandemic but did not do so until after the onset of the pandemic.

To account for potential baseline differences between the two groups, we analyzed baseline equivalence and then used the baseline variables to create inverse-probability of treatment weights (IPTW) to maximize balance on baseline characteristics between the two groups of mothers being compared. These variables also served as covariates in some of the regression models. The baseline variables included: site (i.e., city of recruitment), mother’s age, mother’s education level, household income, mother’s general health, mother’s depressive symptoms, mother’s race and ethnicity, marital status, number of adults in the household, number of other children born to the mother, whether the mother smoked or drank alcohol during pregnancy, whether the child’s father lived in the household, child’s sex, child’s birth weight, and gestational age at birth. Furthermore, we included the child’s age at the Age-1 interview as an additional control.

### 2.3 | Analytic approach

To determine if the experiences of the COVID-19 pandemic and subsequent shelter-in-place orders were associated with mental health and sleep quality, we first had to establish that mothers who completed the interview before the pandemic were similar on observable baseline characteristics to mothers who completed the survey during the pandemic. To compare baseline equivalence between the pre-pandemic and during-pandemic groups at the start of the BFY project in 2018–2019, we estimated a series of OLS regression models that regressed all baseline characteristics on the pandemic onset indicator to determine whether any of the individual baseline variables significantly predicted whether the mothers were interviewed prior to or following the onset of the pandemic. We also conducted a joint test of orthogonality using a multivariate probit model and chi-squared test to determine if the full set of baseline characteristics jointly predicted interview timing.

Following these tests of baseline equivalence, we used the baseline variables to create IPTW weights using an R package called The Toolkit for Weighting and Analysis of Nonequivalent Groups (TWANG), which relies on generalized boosted modeling to maximize equivalence between two groups using a propensity score model (Ridgeway et al., 2017). Generalized boosted modeling is a flexible and nonparametric estimation method that has been shown to outperform other algorithms for propensity score estimation with respect to bias (McCaffrey et al., 2004). The model included all baseline control variables and child’s age at the interview as predictors of whether the mother’s interview took place before or during the COVID-19 pandemic. These weights addressed observable baseline differences between groups.

To test whether the COVID-19 pandemic was significantly associated with differences in depression, anxiety, and sleep quality, we conducted a series of regression analyses to estimate associations between the pandemic-onset indicator and each outcome measure. We estimated three types of models: (1) bivariate OLS regressions that regressed each outcome measure on the pandemic indicator, (2) bivariate OLS regressions with IPTW weights to correct for any observable differences on baseline characteristics, and (3) multivariate OLS regressions that controlled for the baseline covariates.

Finally, we estimated a series of models with interaction terms to analyze whether maternal race and ethnicity, relationship status, and employment history moderated the relationships between the pandemic and maternal depression, anxiety, or sleep quality. Each potential moderator was analyzed using separate models and estimated using the same three modeling approaches described above (i.e., bivariate regression, bivariate regression with IPTW weights, and multivariate regression controlling for baseline covariates).

## 3 | RESULTS

### 3.1 | Sample characteristics

In Table 1, we described the sample characteristics (*n* = 547) using the measures collected during the baseline interview (i.e., at the time of the child’s birth and approximately one year prior to the Age-1 survey). To be eligible for the study, mothers had to have incomes below the poverty line; as expected, the sample was comprised of low-income women who reported an average annual household income of $22,357 in the calendar year prior to their child’s birth. The mean age of the sample was approximately 27 years old, and mothers had on average 2–3 children. Most of the sample identified as Hispanic (42.2%) or Black, non-Hispanic (38.6%). At baseline, most mothers (88.0%) reported good health or better, and the average score on a measure of depressive symptoms (CES-D 10-item short-form) was .68, with about 23.8% of mothers meeting criteria for clinical depression.

### 3.2 | Baseline equivalence

There was adequate balance across all baseline characteristics when comparing the subset of mothers who were interviewed before the pandemic to mothers who were interviewed during the pandemic (see Table A1 in the Appendix). We used a probit model to regress the full set of baseline characteristics on the COVID-19 pandemic indicator to determine whether the baseline characteristics predicted whether the mother was interviewed before or during the COVID-19 pandemic. Using the joint test of orthogonality to test the null hypothesis that all coefficients were equal to zero, the test did not reach the threshold for statistical significance (*p* = .219), which indicated that the two groups of mothers were similar across the observed baseline characteristics. Nevertheless, it is worth noting that a few baseline measures appeared less balanced than others (though not significantly different).

Although there was adequate balance on baseline characteristics, we used generalized boosted models to create inverse-probability treatment weights (IPTW) to ensure that any differences between the pre-pandemic and during-pandemic groups could not be attributed to even minor differences on the baseline characteristics. To examine whether the IPTW weights improved the balance between groups, we calculated effect sizes that captured the standardized mean differences on each baseline characteristic between the pre-pandemic and during-pandemic groups. We reported the maximum and average effect sizes of the baseline differences between the two groups for the unweighted and weighted samples in Table A2 of the Appendix. The IPTW weights successfully reduced the maximum effect size from .23 to .13, and the average effect size decreased from .10 to.06, thereby minimizing the standardized mean differences on baseline characteristics between the two groups.

### 3.3 | Associations between COVID-19 pandemic and depression, anxiety, and sleep quality

On average, mothers who were interviewed during the pandemic reported better mental health and sleep quality than mothers who were interviewed prior to the beginning of the pandemic (Table 2). This was demonstrated by lower average scores for depression and anxiety and higher average sleep quality. Mothers who were interviewed during the pandemic reported about one-point less on both the depression and anxiety scales, equivalent to about one quarter of a standard deviation difference for depression and one-sixth of a standard deviation difference for anxiety. Furthermore, about half as many mothers met criteria for moderate to severe depression and moderate to severe anxiety during the early phase of the pandemic compared to prior to the pandemic. For sleep quality, mothers interviewed after the onset of the pandemic reported 0.53 points higher on the sleep quality scale, which is a difference of about one-fifth of a standard deviation.

Our regression analyses that estimated the associations between the COVID-19 pandemic and each measure of maternal well-being found that the pandemic was most strongly associated with depression (Table 3). Mothers who were interviewed after the onset of the pandemic reported less depression (*p* < .01). This finding was consistent across all three model specifications, with the regression coefficients ranging from −.83 to −1.05. Regression results were more mixed for anxiety and sleep quality. In the bivariate regression and IPTW models, mothers interviewed after the onset of the pandemic reported less anxiety compared to mothers interviewed prior to the pandemic, and this difference was marginally significant (*p* < .10). However, the association was not significant when using multivariate regression to control for baseline covariates. Similarly, the positive association between the pandemic and sleep quality was significant in the bivariate regression model (*p* < .05), marginally significant in the IPTW model (*p* < .10), and not significant in the covariate model.

Given that the interviews were intended to be conducted around the child’s first birthday, mothers who participated when their child was older might be systematically different than mothers who participated closer to the intended interview date. For example, it is possible that a mother who was difficult to reach prior to the pandemic, perhaps due to work constraints, might be more likely to participate after the COVID-19 pandemic created more free time at home. Furthermore, the possibility of participating over the phone rather than having the research team enter the home might have led to differences in who agreed to participate. To address these concerns, we conducted a series of sensitivity checks to see whether the findings were consistent after excluding mothers whose interviews were scheduled late, as determined by the child’s age at the interview. Since all interviews were intended to take place when the child was about 12 months old, these sensitivity tests excluded mothers whose infants were 17 months or older at the time of the Age-1 interview (*n* = 35). After excluding these mothers from the sample, findings were similar. This provided some evidence that delays in retention did not explain the results (see Table A3 in the Appendix).

### 3.4 | Interactions between COVID-19 pandemic and demographic factors

To assess whether the relationship between the COVID-19 pandemic and maternal depression, anxiety, and sleep quality differed by demographic factors, a series of separate moderation analyses were conducted. Three potential moderators were analyzed using separate models for each moderator: relationship status, employment history, and mother’s race and ethnicity. There were no significant interactions between the pandemic indicator and the three potential moderators, suggesting that the associations between the COVID-19 pandemic and maternal depression, anxiety, and sleep quality were consistent regardless of relationship status, employment history, and race/ethnicity (see Table A4 in the Appendix). However, it is important to note that the sample size would have required large and robust differences for significant interactions to be detected.

## 4 | DISCUSSION

Mothers of young children are at an increased risk of experiencing mental health issues and poor sleep quality, and this risk is further elevated under the social and economic conditions associated with poverty. Many scholars, policymakers, and practitioners predicted that the added stressors associated with the COVID-19 pandemic would worsen mental health and sleep quality for mothers raising young children, and several studies have supported this hypothesis. However, in contrast to most of the prior literature on maternal well-being during the pandemic, our study found that low-income mothers of one-year-olds reported lower rates of depression and anxiety and better sleep quality during the early phase of the pandemic compared to before. Across model specifications, the pandemic was significantly associated with depression, providing strong evidence that low-income mothers of one-year-olds experienced less depression during the early phase of the pandemic. The results were less consistent for anxiety and sleep quality. Although there was some evidence that the pandemic was significantly associated with less anxiety and higher sleep quality, the associations were not statistically significant across models. Despite this, even null results are in contrast to prior research that found increases in maternal anxiety during the pandemic. While these results might be surprising given reports of the average mother’s experience during the pandemic, they provide important insights into the unique experiences of the women in this sample, specifically low-income mothers of one-year-olds.

The results of this study demonstrate the importance of paying attention to household context when exploring and discussing maternal mental health. Most of the prior research on maternal mental health during the COVID-19 pandemic was conducted with samples of economically advantaged mothers without considering possible differences by income. In alignment with Silverman et al. (2020), our study found that the early phase of the COVID-19 pandemic might have led to improvements in maternal well-being—particularly lower rates of depression—among low-income mothers of young children. Silverman et al. (2020) found that postpartum mothers with low incomes had lower rates of postpartum depression during the pandemic compared to before. Our study tells a similar story: low-income mothers of one-year-olds reported lower rates of depression and similar if not better anxiety and sleep quality during the first few months of the COVID-19 pandemic.

Future research should examine why low-income mothers fared better during the COVID-19 pandemic than the average mother, particularly in terms of depressive symptoms. Social support is a strong predictor of maternal depression, so it is possible that low-income mothers experienced unexpected increases in social support during this time. For example, the COVID-19 pandemic might have increased the amount of quality time that low-income mothers were able to spend with other household members. This aligns with research finding that some families experienced positive relational effects of the pandemic, including increased connection, decreased conflict, and strengthened relationships (Günther-Bel et al., 2020; Evans et al., 2020). Additionally, these families might have experienced increases in tangible or instrumental support with expansions in social safety net programs, including efforts to make food pantries more accessible, moratoriums on evictions, and expansions in federal programs such as unemployment insurance and food stamps. Positive changes in social support could partially account for decreases in depressive symptoms in our sample.

The developmental age of the children in this study might be especially important to consider when interpreting these results. Mothers in this study had children who were approximately 12 months old at the time of the interview, and it is possible that this is critical for understanding the findings. There might be something unique about having a one-year-old that led the social restrictions to be beneficial for mothers’ well-being. For instance, low-income mothers of one-year-olds might experience more complex work-family stressors related to decisions around work, childcare, breastfeeding, and transportation, and the pandemic may have provided temporary reprieve from some of those stressors. Parenting young children is challenging, especially without adequate supports and resources to help. For this group of mothers, it might have been a relief to have the opportunity to stay home and focus on their young children during this time, especially considering this study took place during the early phase of the pandemic before widespread reports of pandemic parenting burnout and exhaustion set in.

An important caveat to our findings is the timing of our interviews, which occurred in the earliest months of the pandemic. Data collection for this study ended in June 2020. It is possible that any initial improvements in mental health or sleep reported here may have faded or been replaced by negative effects as the income losses, health impacts, parenting burnout, and social isolation persisted beyond the initial phase of the pandemic. However, the social safety net continued to expand as the pandemic persisted, including additional stimulus payments, unemployment benefits, and child tax credits. These additional supports, especially for economically disadvantaged families, might have continued to promote maternal well-being. Regardless, the results of this study must be understood within the context of the timing of our interviews, specifically the early months of the pandemic.

While the current study cannot speak directly to the causal nature of these associations or the mechanisms that potentially led to better mental health and sleep quality for low-income mothers of one-year-olds during the early phase of the pandemic, we can hypothesize potential explanations for these results. As Silverman et al. (2020) discussed, low-income families disproportionately experience social and economic stressors like unavailable childcare, limited partner and family support, and reduced time flexibility—all of which contribute to worse maternal mental health and sleep quality. The social restrictions of the early phase of pandemic might have ameliorated some of those stressors and provided momentary relief for low-income mothers juggling daily demands that exceed their resources. This might have been especially beneficial for mothers’ depressive symptoms. However, it is important to note that potential changes in employment likely also led to reductions in income. While this may have created more economic hardship, it is possible that public investments in the social safety net (e.g., increased unemployment insurance, economic stimulus checks) provided a buffer from the potential harmful effects of income losses. Additionally, there may have been reduced stigma for seeking help during this time and more active support from food pantries and other community resources. Overall, our findings highlight the importance of keeping in mind what pre-pandemic life was like for different groups of mothers when discussing maternal well-being during the pandemic.

### 4.1 | Strengths & limitations

One of the major strengths of this study is the novelty and rigor of the sample, which consists of low-income mothers of one-year-olds who were initially recruited into the larger study prior to the onset of the COVID-19 pandemic. The Baby’s First Years study sample helps minimize concerns regarding sampling bias because it did not rely on convenience sampling; instead, the study sampled mothers within hospitals to allow for a more representative pool of mothers to be enrolled. All the mothers who participated in this study reported incomes below the poverty line when recruited. Therefore, this sample allows us to better understand how low-income mothers of one-year-olds fared during the initial months of the pandemic, which is a unique contribution to the literature.

Additionally, because mothers were enrolled in the study prior to the onset of the pandemic, there were fewer concerns about sampling bias associated with factors related to the COVID-19 pandemic. However, it is still possible that retention was influenced by the COVID-19 pandemic. As mentioned, mothers’ likelihood of participating in the Age-1 interview might have been impacted by the pandemic; for example, the possibility of participating over the phone rather than in-person might have influenced who agreed to participate when. We addressed these concerns by conducting sensitivity tests to see whether the findings were consistent after excluding mothers who were slow to participate, as determined by the child’s age at the interview (i.e., older than 16 months). After excluding those mothers, the findings were mostly similar, which provides evidence that retention delays did not explain the results.

An important limitation of the study design is that it compares different cohorts of women rather than using longitudinal data to examine change over time within the same group of mothers. By examining between-group differences rather than within-group change over time, it is possible that any differences that were detected were due to underlying differences between the two groups rather than due to the COVID-19 pandemic. To alleviate these concerns, we used IPTW to maximize balance on baseline covariates between the two groups. Results were relatively robust to this weighting. However, we must be cautious about interpreting study findings as causally linked to the pandemic given that it is still possible that there were unobserved differences between the two groups that were not controlled.

Due to the protocol changes that went into effect at the start of the pandemic, the mode of interview (i.e., in-person versus phone) was entirely confounded with the pandemic indicator. All mothers in the pre-pandemic group were administered the survey in-person, whereas mothers in the during pandemic group were administered the survey over the phone. Prior research indicates that the modality of phone versus in-person does not affect reports of depression and anxiety (Aneshensel et al., 1982; Paulsen et al., 1988; Rohde et al., 1997). Regardless, it is impossible to entirely dismiss concerns about the mode of interview confounding the results.

Although our depression and anxiety scales (PHQ-8 and BAI, respectively) are validated and commonly used with this population, our measure of sleep quality is a limitation of our study. Our three-item additive index of sleep quality was adapted from the validated PROMIS^™^ Short Form, and this scale has been shown to have greater measurement precision than the Pittsburgh Sleep Quality Index, which is commonly used (Yu et al., 2012). However, we only adapted three of the eight items of the short form, which limits the validity of our measure of sleep quality. Given the paucity of research on self-reported perceptions of sleep quality among mothers of young children during the pandemic, our analyses on sleep quality filled an important gap in the literature, but our specific measure of sleep quality is still a notable limitation.

Our moderation analyses found that demographic factors did not moderate the associations between the pandemic onset and maternal mental health and sleep quality. However, another potential limitation of our study is that the sample size may have been too small to detect small-to-moderate differences in these interaction models. Future studies should continue to explore potential differences in maternal mental health during the pandemic by demographic factors such as relationship status, employment history, and racial and ethnic group.

### 4.2 | Implications for policy

Maternal depression, anxiety, and sleep quality are significant public health issues that disproportionately impact families living in poverty. Our study found lower rates of depression, reduced anxiety, and higher sleep quality among low-income mothers of one-year-olds during the early phase of the pandemic. These results provide an opportunity to re-examine our understanding of how social and economic factors influence maternal well-being, specifically as it relates to families’ abilities to balance demands and resources as they raise young children. For example, despite the added stressors associated with the COVID-19 pandemic, low-income mothers might have experienced relief from some of the daily stressors that typically hinder their mental health and sleep quality. Furthermore, it is possible that the expansions of the social safety net and community resources, including stimulus checks, moratoriums on evictions, and enhanced benefits, may have provided much needed support to these families. Future research should examine the mechanisms that potentially led to more resilience and improvements in maternal mental health and sleep quality among low-income mothers of young children during the early months of the pandemic, especially as it relates to work-family balance and social support. For policymakers and practitioners seeking to promote maternal well-being, this knowledge could provide key insights for ways to improve our social policies and services to better support the quality of life for economically marginalized families moving forward.

## Supplementary Material

Supplemental Appendix

## Figures and Tables

**TABLE 1:** Sample characteristics.

	Mean/(%)	SD
Mother’s age at birth (years)	26.94	(5.84)
Number of children born to mother	2.41	(1.37)
Mother’s health is good or better	88.0%	
Maternal depression (CES-D average score)	0.68	(4.44)
Mother’s education: less than high school diploma	25.1%	
Mother’s education: high school diploma or GED	48.6%	
Mother’s education: some college	17.9%	
Mother’s education: associate degree	3.3%	
Mother’s education: bachelor’s degree or higher	5.1%	
Mother’s race/ethnicity: White, non-Hispanic	10.6%	
Mother’s race/ethnicity: Black, non-Hispanic	38.6%	
Mother’s race/ethnicity: Hispanic, any race	42.2%	
Mother’s race/ethnicity: Multiple or other	8.6%	
Mother is married or cohabitating with partner	48.5%	
Number of cigarettes per week during pregnancy	4.68	(20.26)
Number of alcoholic drinks per week during pregnancy	0.15	(1.66)
Household income	$22,357	($21,280)
Number of adults in the household	2.09	(0.98)
Child’s biological father lives in the household	41.1%	
Child is female	50.6%	
Child’s weight at birth (pounds)	7.13	(1.08)
Child’s gestational age at birth (weeks)	39.09	(1.23)
Child’s age at Age-1 interview (months)	13.14	(2.11)
Site: New Orleans, Louisiana	28.9%	
Site: Twin Cities, Minnesota	11.9%	
Site: Omaha Metro, Nebraska	29.3%	
Site: New York City, New York	30.0%	
*N*	547	

*Note*: All measures were collected during the baseline interview (approximately one year prior to the Age-1 survey), with the exception of child’s age at the Age-1 interview.

**TABLE 2:** Descriptive statistics for depression, anxiety, and sleep quality.

Continuous scales	Full sample	Pre-pandemic	During pandemic
	Mean (SD)	Mean (SD)	Mean (SD)
Depression (PHQ-8)	3.70	4.09	3.03
	(4.01)	(4.17)	(3.63)
Anxiety (BAI)	4.52	4.92	3.85
	(6.32)	(6.47)	(6.01)
Sleep quality	10.77	10.58	11.10
	(2.94)	(2.89)	(2.99)
Clinical categories	%	%	%
Depression (PHQ-8)			
Minimal	68.19%	65.89%	72.06%
Mild	21.94%	21.57%	22.55%
Moderate to severe	9.87%	12.54%	5.39%
Anxiety (BAI)			
Minimal	77.88%	74.34%	83.82%
Mild	14.99%	17.20%	11.27%
Moderate or severe	7.13%	8.45%	4.90%

*Note*: *N* = 547 for depression and anxiety; *N* = 546 for sleep quality.

**TABLE 3:** Associations between the COVID-19 pandemic and depression, anxiety, and sleep quality.

	Depression (PHQ-8)			Anxiety (BAI)			Sleep quality		
	Bivariate regression	Bivariate regression with IPTW	Multivariate Regression	Bivariate Regression	Bivariate Regression with IPTW	Multivariate Regression	Bivariate Regression	Bivariate Regression with IPTW	Multivariate Regression
COVID-19 Pandemic	−1.06**	−.95**	−.83*	−1.06^+^	−.96^+^	−.69	.53*	.51^+^	.43
	(.34)	(.36)	(.32)	(.55)	(.58)	(.51)	(.26)	(.28)	(.27)

*Note*: *N* = 547 for depression and anxiety; *N* = 546 for sleep quality. Depression, anxiety, and sleep quality models were run separately. Standard errors in parentheses. Multivariate regression models controlled for the following baseline characteristics: mother’s age, number of children, mother’s health, mother’s depression, mother’s education, mother’s race, mother’s relationship status, cigarette and alcohol use during pregnancy, household income, number of adults in the home, whether father lives in the home, child’s sex, child’s weight at birth, child’s gestational age at birth, child’s age at interview, and site.

+ *p* < .10,

* *p* < .05,

** *p* < .01.

## Data Availability

The data that support the findings of this study are openly available in ICPSR’s Data Sharing for Demographic Research at https://doi.org/10.3886/ICPSR37871.v2, reference number ICPSR 37871. cd_value_code=text.

## Key Findings

- Low-income mothers of one-year-olds reported better mental health during the early phase of the COVID-19 pandemic compared to before the onset of the pandemic.
- During the pandemic, 5.4% of mothers reported moderate-to-severe depression compared to 12.5% before the pandemic; similarly, 4.9% of mothers reported moderate-to-severe anxiety during the pandemic compared to 8.5% before the pandemic.
- Mothers’ relationship status, employment history, and race/ethnicity did not moderate the associations between the COVID-19 pandemic and depression, anxiety, or sleep quality.

## Statement of Relevance

The COVID-19 pandemic disrupted families’ daily lives. Most research has found that these disruptions took a negative toll on maternal mental health; however, we provide contrasting results among low-income mothers of one-year-olds, suggesting the pandemic differentially impacted families from different economic backgrounds and caregiver circumstances. Maternal well-being is deeply connected to infant mental health, and as we move beyond the pandemic, we must consider how policies and services could better support low-income mothers raising young children.

## Diversity and Anti-Racist Scholarship

The authors take an anti-racist approach to maternal mental health and the circumstances of poverty, acknowledging that each are impacted by socially constructed processes—including structural racism and discrimination. Although this study focuses on the issue of poverty, we recognize that economic resources alone cannot address the social processes and structures that influence well-being for women of color. The authors of this study are majority white and do not reside in the communities represented in the study. Equity, anti-racism, and the diversity of experiences of the participating families are embodied through open conversations with mothers and collaboration with community leaders.

## Footnotes

1 All mothers reported household incomes below the FPL at the time of recruitment into the study; however, we know that household income fluctuates year to year. At the time of the Age-1 survey, 71.4% of mothers reported household incomes below the FPL, 23.2% reported incomes between 100% and 200% of the FPL, and 5.5% reported incomes greater than 200% of the FPL (Gennetian et al., 2022). For this reason, we describe our sample as low-income, which is typically defined as under 200% of the FPL, rather than in poverty.

2 The authors of this study categorized mothers as low socioeconomic status if they met U.S. Government eligibility requirements for Medicaid (i.e., earned less than 138% of the FPL). Because these authors refer to their sample as low socioeconomic status, we also use this terminology while acknowledging that socioeconomic status is more complex than income alone.
