# Supplementary material for: Mental health and sleep quality of low-income mothers of one-year-olds during the COVID-19 pandemic
Source: Infant Ment Health J. Author manuscript; Available in PMC 2023 Aug 4. (PMC10403317; doi:10.1002/imhj.22074)
Supplement: Supplemental Appendix [file NIHMS1917144-supplement-Supplemental_Appendix.docx]

Appendix

| Table A1. Baseline equivalence between pre-pandemic and during pandemic groups | | | | | |
| --- | --- | --- | --- | --- | --- |
|  | Pre-Pandemic (n=342) | During Pandemic (n=205) | Effect Size | | p-value |
|  | Mean (SD) / % | Mean (SD) / % | Hedges' g | Cox's Index |  |
| Mother’s age at birth (years) | 26.7 (5.8) | 27.3 (5.9) | 0.107 |  | 0.315 |
| Number of children born to mother | 2.4 (1.3) | 2.5 (1.5) | 0.051 |  | 0.467 |
| Mother’s health is good or better | 87.4% | 88.8% |  | 0.081 | 0.730 |
| Maternal depression (CESD average score) | 0.7 (0.4) | 0.7 (0.5) | -0.041 |  | 0.832 |
| Mother’s education (years) | 11.9 (2.9) | 11.8 (2.6) | -0.057 |  | 0.462 |
| Race/ethnicity: White, non-Hispanic | 12.3% | 7.8% |  | -0.306 | 0.228 |
| Race/ethnicity: Black, non-Hispanic | 38.9% | 38.0% |  | -0.023 | 0.869 |
| Race/ethnicity: Hispanic, any race | 39.2% | 47.3% |  | 0.200 | 0.114 |
| Race/ethnicity: Multiple or other | 9.6% | 6.8% |  | -0.227 | 0.435 |
| Mother is married or cohabitating | 47.7% | 50.2% |  | 0.061 | 0.516 |
| Cigarettes per week during pregnancy | 5.3 (21.7) | 3.7 (17.8) | -0.077 |  | 0.553 |
| Alcohol drinks per week during pregnancy | 0.0 (0.5) | 0.3 (2.6) | 0.174 |  | 0.133 |
| Household income | $22,892 ($20,558) | $21,446 ($22,482) | -0.068 |  | 0.641 |
| Number of adults in the household | 2.1 (1.0) | 2.0 (0.9) | -0.116 |  | 0.130 |
| Child’s biological father lives in household | 39.5% | 43.9% |  | 0.110 | 0.313 |
| Child is female | 50.9% | 50.2% |  | -0.017 | 0.772 |
| Child’s weight at birth (pounds) | 7.1 (1.0) | 7.2 (1.1) | 0.150 |  | 0.069 |
| Child’s gestational age at birth (weeks) | 39.0 (1.3) | 39.2 (1.1) | 0.141 |  | 0.070 |
| Joint Test: Chi^2^ = 23.43, p-value= 0.219, n=547. | | | | | |

*Note.* All measures were collected during the baseline interview, approximately one year prior to the Age-1 survey. P-values were derived from a series of OLS bivariate regressions in which each respective baseline characteristic was regressed on a pandemic-onset indicator using robust standard errors and site-level fixed effects. The joint test of orthogonality was conducted using a probit model with robust standard errors and site-level fixed effects. Standardized mean differences were calculated using Hedges’ g for continuous measures and Cox’s Index for dichotomous measures.

| Table A2. Effect sizes of baseline differences between pre-pandemic and during pandemic groups |
| --- |

|  | Maximum effect size | Average effect size |
| --- | --- | --- |
| Unweighted | 0.23 | 0.10 |
| IPTW | 0.13 | 0.06 |

*Note.* Effect sizes represent the degree to which baseline characteristics were associated with whether the Age-1 interview took place before or during the COVID-19 pandemic. Unweighted effect sizes were calculated prior to implementing the IPTW weights; IPTW effect sizes use the weights to adjust for baseline differences between groups.

| Table A3. Associations between COVID-19 pandemic and depression, anxiety, and sleep quality after excluding children older than 16 months at the time of the interview. | | | | | | | | | |
| --- | --- | --- | --- | --- | --- | --- | --- | --- | --- |
|  | Depression (PHQ8) | | | Anxiety (BAI) | | | Sleep Quality | | |
|  | Bivariate Regression | Bivariate Regression with IPTW | Multivariate Regression | Bivariate Regression | Bivariate Regression with IPTW | Multivariate Regression | Bivariate Regression | Bivariate Regression with IPTW | Multivariate Regression |
| COVID-19 Pandemic | -1.10** | -0.95* | -0.85* | -1.24* | -1.06^+^ | -0.78 | 0.52^+^ | 0.50^+^ | 0.45 |
|  | (0.36) | (0.37) | (0.34) | (0.56) | (0.60) | (0.52) | (0.27) | (0.29) | (0.28) |
| Observations | 512 | 512 | 512 | 512 | 512 | 512 | 511 | 511 | 511 |

*Note.* Depression, anxiety, and sleep quality models were run separately. These models excluded all children who were greater than 16 months old at the time of the Age-1 interview (n=35). Standard errors in parentheses. Multivariate regression models controlled for the following baseline characteristics: mother’s age, number of children, mother’s health, mother’s depression, mother’s education, mother’s race, mother’s relationship status, cigarette and alcohol use during pregnancy, household income, number of adults in the home, whether father lives in the home, child’s sex, child’s weight at birth, child’s gestational age at birth, child’s age at interview, and site. ^+^ *p* < 0.10, ^*^ *p* < 0.05, ^**^ *p* < 0.01

| Table A4. Main effects and interaction terms from moderation models. | | | |
| --- | --- | --- | --- |
|  | Depression (PHQ8) | Anxiety (BAI) | Sleep Quality |
| Relationship Status |  |  |  |
| COVID-19 Pandemic | -0.99* | -1.69* | 0.69+ |
|  | (0.49) | (0.75) | (0.38) |
| Married or cohabitating | -0.31 | -0.50 | 0.39 |
|  | (0.47) | (0.74) | (0.32) |
| COVID-19 X Married or cohabitating | -0.31 | 0.80 | -0.23 |
|  | (0.69) | (1.11) | (0.52) |
| Employment Status |  |  |  |
| COVID-19 Pandemic | 1.72** | -2.53* | 0.88+ |
|  | (0.65) | (1.04) | (0.46) |
| Employed before pregnancy | -0.98 | -1.85+ | 0.34 |
|  | (0.64) | (1.03) | (0.43) |
| COVID-19 X Employed before pregnancy | 0.69 | 1.61 | -0.28 |
|  | (0.82) | (1.34) | (0.60) |
| Race/Ethnicity |  |  |  |
| COVID-19 | -1.66 | 0.89 | 0.82 |
|  | (1.26) | (2.85) | (0.95) |
| Black, non-Hispanic | -1.98* | -3.44** | 1.03+ |
|  | (0.86) | (1.19) | (0.56) |
| Hispanic | 2.55** | -3.60** | 1.22* |
|  | (0.86) | (1.18) | (0.62) |
| COVID-19 X Black, non-Hispanic | 0.95 | -1.95 | -0.25 |
|  | (1.38) | (2.86) | (1.04) |
| COVID-19 X Hispanic | 0.68 | -1.21 | -0.36 |
|  | (1.34) | (2.88) | (1.02) |
| Observations | 547 | 547 | 546 |

*Note.* Standard errors in parentheses. Depression, anxiety, and sleep quality models were runs separately. All models used IPTW weights to account for baseline differences between COVID-19 pandemic groups. ^+^ *p* < 0.10, ^*^ *p* < 0.05, ^**^ *p* < 0.01
